# *FTO* Obesity Risk Variants Are Linked to Adipocyte *IRX3* Expression and BMI of Children - Relevance of *FTO* Variants to Defend Body Weight in Lean Children?

**DOI:** 10.1371/journal.pone.0161739

**Published:** 2016-08-25

**Authors:** Kathrin Landgraf, Markus Scholz, Peter Kovacs, Wieland Kiess, Antje Körner

**Affiliations:** 1 Center for Pediatric Research Leipzig (CPL), University Hospital for Children & Adolescents, University of Leipzig, Leipzig, Germany; 2 Integrated Research and Treatment Center (IFB) Adiposity Diseases, University of Leipzig, Leipzig, Germany; 3 Institute for Medical Informatics, Statistics and Epidemiology, University of Leipzig, Leipzig, Germany; 4 LIFE Research Center for Civilization Diseases, University of Leipzig, Leipzig, Germany; GDC, GERMANY

## Abstract

**Background:**

Genome-wide association studies have identified variants within the *FTO* (fat mass and obesity associated) locus as the strongest predictors of obesity amongst all obesity-associated gene loci. Recent evidence suggests that variants in *FTO* directly affect human adipocyte function through targeting *IRX3* and *IRX5* and thermogenesis regulation.

**Aim:**

We addressed the relevance of this proposed *FTO-IRX* pathway in adipose tissue (AT) of children.

**Results:**

Expression of *IRX3* was higher in adipocytes compared to SVF. We found increased adipocyte-specific expression of *IRX3* and *IRX5* with the presence of the *FTO* risk haplotype in lean children, whereas it was unaffected by risk variants in obese peers. We further show that *IRX3* expression was elevated in isolated adipocytes and AT of lean compared to obese children, particularly in *UCP1*-negative adipocytes, and inversely correlated with BMI SDS. Independent of BMI, *IRX3* expression in adipocytes was significantly related to adipocyte hypertrophy, and subsequent associations with AT inflammation and HOMA-IR in the children.

**Conclusion:**

One interpretation of our observation of *FTO* risk variants linked to *IRX3* expression and adipocyte size restricted to lean children, along with the decreased *IRX3* expression in obese compared to lean peers, may reflect a defense mechanism for protecting body-weight, which is pertinent for lean children.

## Introduction

Genetic variants in the *FTO* (fat mass and obesity associated) gene have been discovered and repeatedly confirmed to pose the strongest (poly)genetic risk for human obesity [1–3]. Multiple studies have focused on deciphering potential mechanisms, by which variants within a region of high linkage disequilibrium in introns 1 and 2 of *FTO* confer the obesity risk.

Initial observations that fasting is associated with decreased hypothalamic *Fto* expression and that manipulations in hypothalamic *Fto* expression affect food intake in mice [4] suggested a central mode of action for *FTO*. In humans, presence of the risk genotype only subtly modulated the amount and/or the preferences of ingested food [5, 6]. Studies in *Fto* knockdown mice showing reduced adipose tissue (AT) mass despite hyperphagia or unaltered food intake but increased energy expenditure [7, 8] questioned whether the susceptibility alleles and *FTO* convey their effects entirely in the brain and point to the adipose tissue itself as a potential target. Very recently, Claussnitzer *et al*. delineated a new mechanism whereby variants in the *FTO* gene affect human adipocyte function through targeting *IRX3* and *IRX5* [9]. The potential functional link between *FTO* and *IRX3* is supported by evidence from murine, human, and *in vitro* studies showing that the *IRX3* promoter strongly interacts with the obesity-associated interval within *FTO*, and that obesity-linked SNPs are associated with *IRX3* but not with *FTO* expression [10, 11]. Knockout of *Irx3* in mice led to a significant reduction in body weight, increased activation of brown AT and increased energy expenditure [11]. In the human study, the causal rs1421085 variant resulted in the activation of *IRX3* and *IRX5* expression during early adipocyte differentiation and a developmental shift from energy-dissipating beige adipocytes to energy-storing white adipocytes [9] and thereby may contribute to the predisposition for obesity, even though the study was restricted to lean healthy adults.

Within this study, we aimed to assess whether the link between the *FTO* risk genotype and *IRX3* and *IRX5* expression in AT is related to the development of obesity and to alterations of AT biology in children.

## Materials and Methods

### Subjects and samples (Leipzig Childhood Adipose Tissue cohort)

Subcutaneous AT samples from 45 lean and 47 overweight and obese children included in the previously described Leipzig Childhood Adipose Tissue cohort [12] were obtained during elective surgery. Children underwent detailed anthropometric, clinical and metabolic assessments [12]. The study was approved by the ethics committee of the Medical Faculty, University of Leipzig (Reg.No: 265-08-ff; NCT02208141) and written informed consent was obtained from all parents.

Preparation of and analyses of AT samples was performed according to previously published protocols [12]. Briefly, adipocytes and stromal vascular fraction (SVF) were isolated by collagenase digestion and adipocyte diameter was determined after osmium fixation using a Coulter counter (Multisizer III; Beckmann Coulter). Macrophage infiltration was analysed by immunohistochemical staining of AT sections with CD68 antibody (M0718, DAKO).

Prior to surgery, fasting blood samples were obtained and stored at -80°C. Analyses of serum parameters (adiponectin, leptin, high sensitivity C-reactive protein (hsCRP), glucose and insulin) were performed by a certified laboratory (Institute of Laboratory Medicine, Clinical Chemistry and Molecular Diagnostics, University of Leipzig).

### DNA-Isolation and genotyping

DNA from EDTA blood samples was isolated using the QIAmp DNA Blood MiniKit (Qiagen). Samples were genotyped as part of the LIFE Child study (43 plates comprising 4,128 samples) using the Affymetrix Axiom Genome-Wide CEU 1 Array. Sample quality control included dish-qc> = 0.82, call rate> = 97%, sex-mismatches, implausible relatedness and outliers of principal components analysis. Duplicates and controls were removed resulting in a total of 3,797 successfully genotyped samples. SNP quality control was passed with cluster plot criteria of Affymetrix, call rate ≥97%, p-value of Hardy-Weinberg disequilibrium test >10^−6^ and plate-association p-value >10^−7^ in 541.835 autosomal SNPs. Data were imputed on the 1000Genomes reference panel (phase 1, release V3 of CEU, HG19, dbSNP-build 135) using SHAPEIT Version v2.r778 with standard settings for European populations and IMPUTE2, version 2.3.0.

According to Claussnitzer *et al*., participants were grouped into risk-, heterozygous, or nonrisk-allele carries depending on their genotype for the *FTO* obesity variants rs9930506, rs1421085 (directly genotyped) and rs1558902 (imputed) [9].

### RNA isolation and mRNA expression analyses

RNA isolation and cDNA synthesis from whole AT samples, isolated adipocytes or SVF cells was performed as previously described [12]. *IRX3* and *IRX5* expression levels were determined by quantitative *real-time* RT-PCR using SYBR green. *UCP1* expression was determined as described [13]. Expression levels were normalized to the reference genes *TBP*, *ACTB* and *HPRT* [14]. Primer and probe sequences are listed in S1 Table.

### Protein isolation and immunoblot analyses

Protein isolation was performed using TRIzol Reagent (Thermo Fisher Scientific) according to the manufacturer’s instructions. Equivalent amounts of proteins were resolved by 10% SDS-PAGE and immunoblotting using antibodies against IRX3 (abcam, ab174307) and beta-ACTIN (abcam, ab8227).

### Statistical analyses

All statistical analyses were performed using the Statistica 10.0 software package (StatSoft). Before analyses, normal distribution of the data was assessed by Shapiro-Wilks W test and quantile quantile plots. Non-normally distributed data were log-transformed before analyses. Quantitative traits were analysed using parametric tests (Pearson correlation analysis, Student's *t* test, one-way ANOVA with Dunnett’s post-hoc test). Categorical variables were analysed by chi square test. For statistical analyses, overweight and obese children were combined. For multiple regression analyses, the stepwise forward model was employed.

## Results

General characteristics of patients and samples of the Leipzig Childhood AT cohort included in this study are summarized in Table 1. As previously described we detected characteristic obesity-related alterations in AT and serum parameters, such as hypertrophy and inflammation [12].

**Table 1:** Characteristics of the Childhood Adipose Tissue Cohort (n = 92).

		Lean			Obese		
	n	Mean±SEM	Range	n	Mean±SEM	Range	*p*
*Anthropometric parameters*							
Male/Female (% male)		20/25 (44.4)			18/29 (38.3)		0.549
Age [years]	45	10.0±0.8	1.1–18.3	47	13.3±0.5	4.8–18.4	**<0.001**
PH	40	2.5±0.3	1–6	45	3.4±0.2	1–6	**0.011**
BMI SDS	45	-0.1±0.1	-2.4–1.1	47	2.5±0.1	1.4–4.3	**<0.001**
*Adipose tissue parameters*							
Adipocyte diameter [μm]	25	111.9±2.4	90.9–131.2	33	125.8±2.4	98.0–146.2	**<0.001**
*Macrophage infiltration*							
Macrophages per 100 adipocytes	37	11.1±1.4	0–29	39	20.5±3.4	0–115	**0.031**^a^
*CD68* mRNA in SVF	36	0.6±0.1	0.1–1.7	32	1.3±0.2	0.0–3.8	**<0.001**^a^
*Serum parameters*							
Adiponectin [mg/l]	34	10.9±1.3	3.7–43.8	38	5.7±0.4	1.7–11.7	**<0.001**^a^
Leptin [ng/ml]	30	5.1±0.8	0.4–17.5	38	31.0±3.3	1.3–83.6	**<0.001**^a^
hsCRP [mg/l]	34	0.6±0.1	0.3–3.2	38	1.9±0.3	0.3–9.9	**<0.001**^a^
HOMA-IR	34	1.4±0.2	0.1–5.6	37	4.0±0.3	0.6–8.2	**<0.001**^a^
*Genetic parameters*							
*FTO* haplotype(nonrisk/heterozygous/risk)		14/14/7			7/16/11		0.188

Data are given as mean ± SEM. For gender and *FTO* haplotype, statistical significance was analysed by chi square test. Statistical significance for differences between groups was determined by Students *t*-test. Significant *p*-values are indicated in bold. PH, pubertal stage; BMI, body-mass index; SDS, standard deviation score; SVF, stroma-vascular fraction; hsCRP, high sensitivity C-reactive protein; HOMA-IR, homeostasis model assessment of insulin resistance.

^a^Statistical analyses were performed for log-transformed parameters.

**Fig 1:**
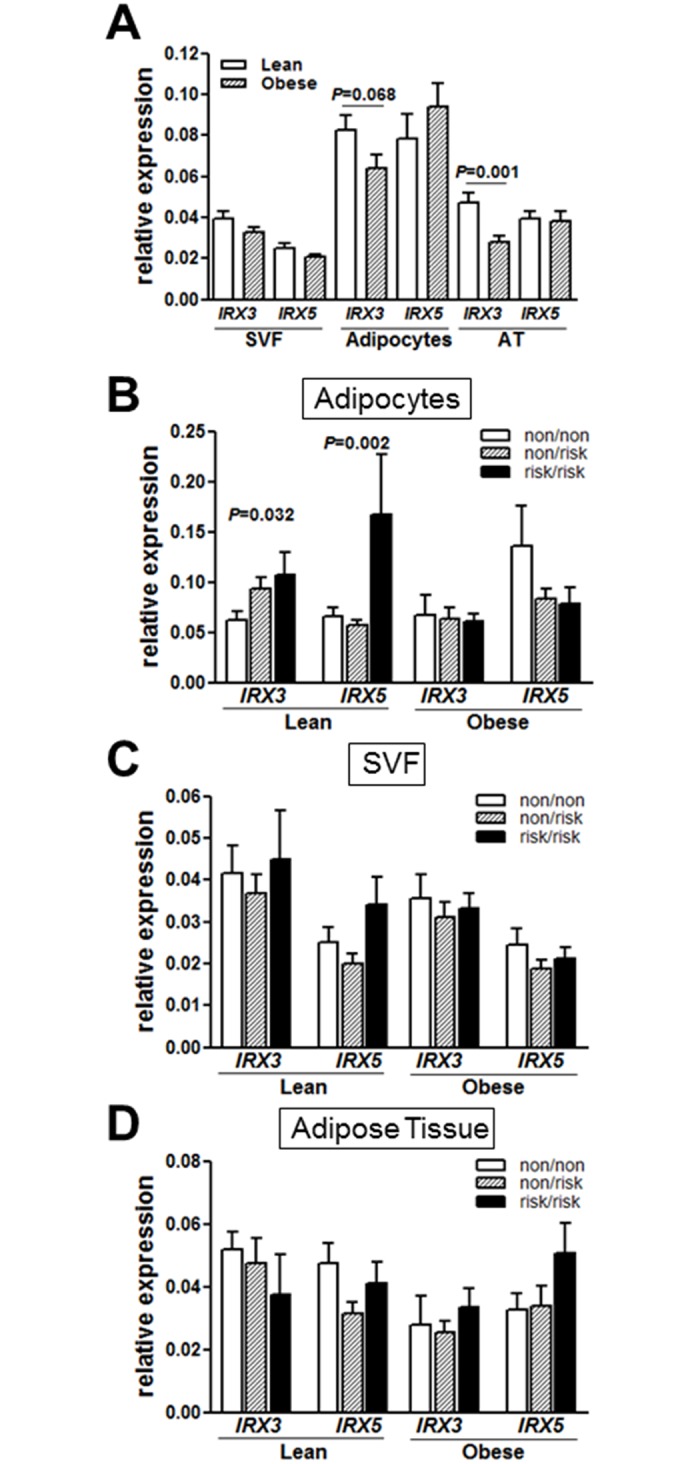
Expression of *IRX3* and *IRX5* in adipose tissue of children and association with *FTO* risk haplotype and with *UCP1* expression. Adipocyte and whole AT expression of *IRX3* was reduced in obese compared to lean children (A). Adipocyte-specific expression of *IRX3* and *IRX5* was significantly increased in lean children carrying the *FTO* risk haplotype compared to lean children carrying the nonrisk haplotype (B). There were no alterations in *IRX3* and *IRX5* expression between *FTO* haplotype groups in SVF (C) or whole AT (D) of lean children. Obese children did not show differences in *IRX3* and *IRX5* expression levels between *FTO* haplotype groups in any of the analyzed tissues (B-D). Significant differences between *FTO* nonrisk versus risk haplotype groups were assessed by Student’s *t*-test and significant *p*-values are indicated in the barplots.

We first evaluated potential differences in *IRX3* and *IRX5* expression in lean and obese children. Considering that obese children are slightly older compared to lean children and, hence, show a more advanced pubertal stage, we analyzed the effect of pubertal stage on *IRX3* and *IRX5* expression in SVF cells, adipocytes and whole AT of lean children. We did not detect significant puberty-related alterations in *IRX3* and *IRX5* expression (S1 Fig). If *FTO* variants confer obesity risk by driving the expression of *IRX3* [9], one may expect increased expression of *IRX* genes in obese subjects. However, *IRX3* expression was unchanged in SVF of obese children and appeared to be even lower in whole adipose tissue samples and isolated adipocytes of obese compared to lean children (Fig 1A). *IRX5* expression was not different between lean and obese children. Concordantly, adipocyte *IRX3* expression negatively correlated with BMI SDS (R = -0.265, *P* = 0.016), which was not found for *IRX5*.

As this observation may point to potentially distinct regulation in AT subfractions, we compared *IRX3* and *IRX5* expression in whole adipose tissue and freshly isolated SVF cells and adipocytes. We detected highest expression of both *IRX3* and *IRX5* in adipocytes (Fig 1A).

To evaluate whether the *IRX* expression might be genetically driven by *FTO* risk variants, we next assessed the expression of *IRX3* and *IRX5* according to *FTO* risk haplotype. Only in the lean subcohort, adipocyte-specific expression of both *IRX3* and *IRX5* was increased in risk-allele carriers compared to nonrisk-allele carriers, which was not observed in obese children (Fig 1B) similar to what was found in lean adults [9]. In line with Claussnitzer *et al*. we did not detect differences in *IRX3* and *IRX5* expression between genotype groups in whole AT or SVF (Fig 1C and 1D).

**Fig 2:**
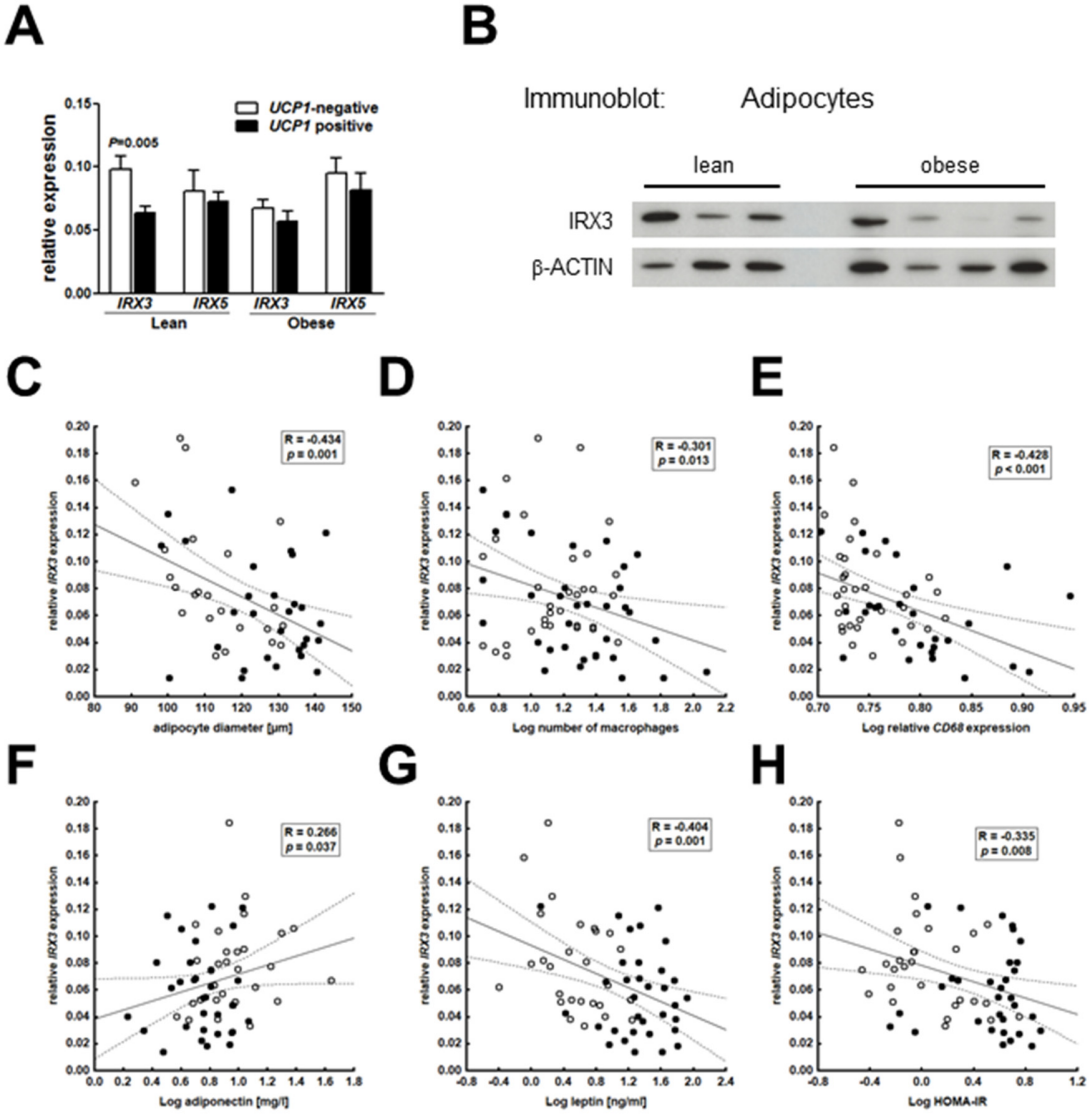
Adipocyte *IRX3* expression is associated with obesity-related alterations in AT and related clinical traits. Expression of *IRX3* but not *IRX5* was significantly lower in *UCP1*-positive (18 lean, 19 obese) compared *UCP1*-negative adipocytes (21 lean, 23 obese) derived from lean, but not of obese children (A). Significant differences between *UCP1*-positive versus *UCP1*-negative adipocyte samples were assessed by Student’s *t*-test and *p*-values are indicated in the barplots. Protein levels of IRX3 are reduced in adipocyte of obese (n = 4) compared to lean (n = 3) children (B). Expression of *IRX3* in adipocytes of children was associated with adipocyte diameter (C), macrophage infiltration as indicated by the number of adipocytes in AT (D) and *CD68* expression in the stroma-vascular fraction (E), adiponectin (F) and leptin (G) serum levels, as well as HOMA-IR as a measure insulin resistance (H). Pearson correlation coefficient R and *p*-value are given in each scatter plot. Significant *p*-values (*p*<0.05) are indicated in bold.

Considering that *IRX3* and *IRX5* expression were directly linked to mitochondrial thermogenesis and negatively associated with the expression of thermogenic genes [9], we were interested in the existence of a similar link in adipocytes of children. When we stratified our samples into *UCP1*-negative and *UCP1*-positive (depending on detectable *UCP1* expression), we found increased expression of *IRX3* in *UCP1*-negative compared to *UCP1*-positive adipocytes of lean children, while there was no difference in *IRX3*-expression in obese children (Fig 2A). Again, we did not find any alterations in *IRX5* expression according to *UCP1*-expression in lean nor in obese children (Fig 2A). Quantitatively, neither *IRX3* nor *IRX5* expression correlated with *UCP1* expression in *UCP1*-positive adipocytes.

Finding *IRX3* mRNA (Fig 1A) and protein (Fig 2B) expression decreased in adipocytes of obese children, we further evaluated whether *IRX3* expression may also be related to obesity related AT alterations. Indeed, *IRX3* expression in adipocytes negatively correlated with obesity-related parameters of AT dysfunction, i.e. adipocyte diameter (Fig 2C) and AT inflammation as indicated by the number of infiltrating macrophages (Fig 2D) and *CD68* mRNA expression in the SVF (Fig 2E). These correlations withstood adjustment for BMI SDS and age of children in partial correlation analysis (adipocyte diameter: R_adj_ = -0.358, *p* = 0.006; macrophage number: R_adj_ = -0.254, *p* = 0.041; *CD68* expression in SVF: R_adj_ = -0.371, *p* = 0.008). Moreover, we detected significant associations of adipocyte *IRX3* expression with adiponectin (Fig 2F) and leptin (Fig 2G) serum levels and with HOMA-IR (Fig 2H) as a measure of insulin resistance, which were, however, lost after adjustment for BMI SDS and age. All of these correlations were independent of pubertal development, which has a major impact on insulin resistance (S2 Table).

Finally, we performed multiple regression analyses to determine the main predictors for adipocyte *IRX3* and *IRX5* expression in AT of children. Adipocyte diameter and the *FTO* risk haplotype independently determined adipocyte *IRX3* expression accounting for 12 and 6% of variability. For *IRX5* expression, the *FTO* risk haplotype was not predictive (Table 2).

**Table 2:** Multiple regression analyses for predictors of adipocyte *IRX3/5* expression.

Step	Parameter	Delta R^2^	Beta ± SEM	*p*
*independent variables for all models*:				
*age*, *gender*, *PH*, *BMI SDS*, *adipocyte diameter*, *Log macrophage number*, *FTO haplotype*				
*dependent variable*: ***IRX3 expression in adipocytes*** *(R*^*2*^ = *0*.*30*, *p = 0*.*002*, *n = 44)*				
1	adipocyte diameter	0.12	-0.42±0.14	**0.005**
2	*FTO* haplotype	0.06	-0.29±0.14	**0.038**
3	Log macrophage number	0.12	-0.23±0.14	0.110
*dependent variable*: ***IRX5 expression in adipocytes*** *(R*^*2*^ = *0*.*26*, *p = 0*.*019*, *n = 44)*				
1	adipocyte diameter	0.39	-0.48±0.18	**0.010**
2	BMI SDS	0.33	0.37±0.17	**0.035**
3	age	0.20	0.28±0.15	0.073
4	Log macrophage number	0.11	-0.16±0.15	0.273

PH, pubertal stage; BMI, body-mass index; SDS, standard deviation score. Significant *p*-values (*p*<0.05) are indicated in bold.

## Discussion

In this study, we have addressed the relevance of the proposed *FTO-IRX* pathway in AT samples of children. Our findings of increased adipocyte-specific expression of *IRX3* and *IRX5* with the presence of the *FTO* risk haplotype in lean individuals not only complement those of the previous study, which was restricted to lean adults. Moreover, we show higher expression levels of *IRX3* and *IRX5* in adipocytes compared to SVF and we further show that *IRX3* expression is elevated in AT and isolated adipocytes of lean compared to obese children and negatively correlates with BMI SDS. Independent of BMI, *IRX3* expression in adipocytes was significantly related to adipocyte hypertrophy, which may explain subsequent associations with AT inflammation and parameters of insulin resistance.

Claussnitzer *et al*. performed studies in adipocytes of lean adult humans and delineated the activation of two genes near the *FTO* locus, *IRX3* and *IRX5*, during early adipocyte differentiation as a new mechanism conferring genetic obesity risk whereby variants in *FTO* directly affect adipocyte function [9]. We provide evidence that this link between *FTO* risk variants and *IRX3/5* expression is already active in children. Nevertheless, this proposed *FTO-IRX* association may be restricted to lean subjects as indicated by increased *IRX3* and *IRX5* expression in adipocytes of lean children (and adults) carrying the *FTO* risk haplotype, whereas it was unaffected by risk variants in obese children. In contrast to our study, previous studies did not analyze *IRX* expression or a potential association of *FTO* risk variants with *IRX* expression in AT of obese patients. It would, hence, be interesting whether the proposed *FTO-IRX* association is restricted to lean adults similar to what we have seen in children.

According to Claussnitzer *et al*., enhanced expression of *IRX3* and *IRX5* results in a shift from white adipocyte browning to lipid storage [9]. In line with these data we observed increased *IRX3* expression in *UCP1*-negative adipocytes compared to *UCP1*-positive adipocytes, but we did not observe an association of *IRX5* and *UCP1* expression in adipocytes of children indicating that *IRX3* might be the main mediator of obesity risk in *FTO* risk variant carriers in children.

Concluding from the observed association of *IRX3* expression with *FTO* obesity risk variants and *UCP1* expression in adipocytes of lean children, one may expect increased *IRX3* expression in obese subjects. However, *IRX3* expression was even lower in isolated adipocytes of obese compared to lean children and correlated with obesity-related measures of adipocyte hypertrophy and inflammation. Interestingly, adipocyte diameter was the strongest independent predictor of adipocyte *IRX3* expression. An interpretation for this and Claussnitzer´s finding of an *IRX*-dependent shift from energy consumption to energy storage in adipocytes may be the protection of body weight under circumstances of limited energy supply. Similar hypotheses have been discussed for downregulation of leptin levels to signal energy insufficiency [15]. Interestingly, obesity-associated *FTO* risk alleles have been discussed as candidate thrifty alleles, which have been driven to high frequency by positive selection [16]. According to the thrifty gene hypothesis, populations whose ancestral environments were characterized by alternating periods of food abundance and food shortage experienced positive selection for alleles that promote storage of fat in order to provide a survival advantage [17]. Such defense mechanisms for preserving body weight would be pertinent for lean subjects, particularly children, and may be attenuated in the obese state.

One strength of our study is that we provide data on whole AT as well as freshly isolated SVF cells and adipocytes of humans, which might be closer to physiological conditions compared to analyses performed in cell cultures of primary cells. However, we are limited by the often small sample volumes in children, which precluded more mechanistic analyses.

In conclusion, our results indicate a relationship between *FTO* variants and *IRX3* expression and adipocyte phenotype in lean children, which is attenuated in the obese state.

## Supporting Information

S1 Fig Expression of *IRX3* in AT of lean children and association with puberty stage. There were no significant differences in *IRX3* and *IRX5* expression in SVF cells, adipocytes or AT between pre-pubertal, pubertal and post-pubertal children. Differences between puberty stages were assessed by one-way ANOVA and Dunnett’s post-hoc test. A *P*-value of less than 0.05 was considered significant. SVF, stroma-vascular fraction. AT, adipose tissue.
(TIF)

S1 Table Primer and probe sequences for quantitative *real-time* RT-PCR. (DOC)

S2 Table Comparison of unadjusted and puberty-adjusted correlations of adipocyte *IRX3* with obesity-related patient parameters. (DOC)

## References

[1] Dina C, Meyre D, Gallina S, Durand E, Körner A, Jacobson P, et al Variation in FTO contributes to childhood obesity and severe adult obesity. Nature genetics. 2007;39(6):724–6. 10.1038/ng2048 . 17496892

[2] Frayling TM, Timpson NJ, Weedon MN, Zeggini E, Freathy RM, Lindgren CM, et al A common variant in the FTO gene is associated with body mass index and predisposes to childhood and adult obesity. Science. 2007;316(5826):889–94. 10.1126/science.1141634 17434869 PMC2646098

[3] Loos RJ, Yeo GS. The bigger picture of FTO: the first GWAS-identified obesity gene. Nature reviews Endocrinology. 2014;10(1):51–61. 10.1038/nrendo.2013.227 24247219 PMC4188449

[4] Tung YC, Ayuso E, Shan X, Bosch F, O'Rahilly S, Coll AP, et al Hypothalamic-specific manipulation of Fto, the ortholog of the human obesity gene FTO, affects food intake in rats. PloS one. 2010;5(1):e8771 10.1371/journal.pone.0008771 20098739 PMC2808248

[5] Qi Q, Downer MK, Kilpelainen TO, Taal HR, Barton SJ, Ntalla I, et al Dietary Intake, FTO Genetic Variants, and Adiposity: A Combined Analysis of Over 16,000 Children and Adolescents. Diabetes. 2015;64(7):2467–76. 10.2337/db14-1629 . 25720386 PMC4876751

[6] Cecil JE, Tavendale R, Watt P, Hetherington MM, Palmer CN. An obesity-associated FTO gene variant and increased energy intake in children. The New England journal of medicine. 2008;359(24):2558–66. 10.1056/NEJMoa0803839 . 19073975

[7] Church C, Moir L, McMurray F, Girard C, Banks GT, Teboul L, et al Overexpression of Fto leads to increased food intake and results in obesity. Nature genetics. 2010;42(12):1086–92. 10.1038/ng.713 21076408 PMC3018646

[8] Fischer J, Koch L, Emmerling C, Vierkotten J, Peters T, Brüning JC, et al Inactivation of the Fto gene protects from obesity. Nature. 2009;458(7240):894–8. 10.1038/nature07848 . 19234441

[9] Claussnitzer M, Dankel SN, Kim KH, Quon G, Meuleman W, Haugen C, et al FTO Obesity Variant Circuitry and Adipocyte Browning in Humans. The New England journal of medicine. 2015;373(10):895–907. 10.1056/NEJMoa1502214 . 26287746 PMC4959911

[10] Ragvin A, Moro E, Fredman D, Navratilova P, Drivenes O, Engstrom PG, et al Long-range gene regulation links genomic type 2 diabetes and obesity risk regions to HHEX, SOX4, and IRX3. Proceedings of the National Academy of Sciences of the United States of America. 2010;107(2):775–80. 10.1073/pnas.0911591107 20080751 PMC2818943

[11] Smemo S, Tena JJ, Kim KH, Gamazon ER, Sakabe NJ, Gomez-Marin C, et al Obesity-associated variants within FTO form long-range functional connections with IRX3. Nature. 2014;507(7492):371–5. 10.1038/nature13138 24646999 PMC4113484

[12] Landgraf K, Rockstroh D, Wagner IV, Weise S, Tauscher R, Schwartze JT, et al Evidence of early alterations in adipose tissue biology and function and its association with obesity-related inflammation and insulin resistance in children. Diabetes. 2015;64(4):1249–61. 10.2337/db14-0744 . 25392242

[13] Rockstroh D, Landgraf K, Wagner IV, Gesing J, Tauscher R, Lakowa N, et al Direct evidence of brown adipocytes in different fat depots in children. PloS one. 2015;10(2):e0117841 10.1371/journal.pone.0117841 25706927 PMC4338084

[14] Bernhard F, Landgraf K, Kloting N, Berthold A, Buttner P, Friebe D, et al Functional relevance of genes implicated by obesity genome-wide association study signals for human adipocyte biology. Diabetologia. 2013;56(2):311–22. 10.1007/s00125-012-2773-0 . 23229156

[15] Flier JS. Clinical review 94: What's in a name? In search of leptin's physiologic role. The Journal of clinical endocrinology and metabolism. 1998;83(5):1407–13. 10.1210/jcem.83.5.4779 . 9589630

[16] Stratigopoulos G, Martin Carli JF, O'Day DR, Wang L, Leduc CA, Lanzano P, et al Hypomorphism for RPGRIP1L, a ciliary gene vicinal to the FTO locus, causes increased adiposity in mice. Cell metabolism. 2014;19(5):767–79. 10.1016/j.cmet.2014.04.009 24807221 PMC4131684

[17] Friedman JM. A war on obesity, not the obese. Science. 2003;299(5608):856–8. 10.1126/science.1079856 . 12574619

