# Supplementary figures and images for: FTO Obesity Risk Variants Are Linked to Adipocyte IRX3 Expression and BMI of Children - Relevance of FTO Variants to Defend Body Weight in Lean Children?
Source: PLoS One. 2016 Aug 25;11(8):e0161739. doi: 10.1371/journal.pone.0161739 (PMC4999231; doi:10.1371/journal.pone.0161739)

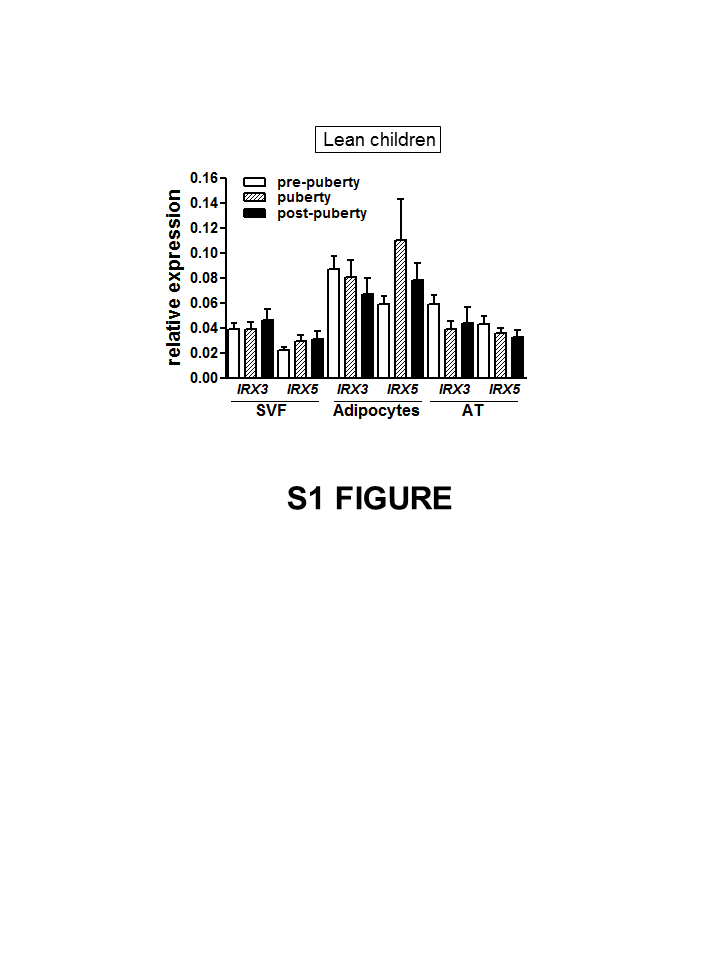

Supplement: S1 Fig — There were no significant differences in IRX3 and IRX5 expression in SVF cells, adipocytes or AT between pre-pubertal, pubertal and post-pubertal children. Differences between puberty stages were assessed by one-way ANOVA and Dunnett’s post-hoc test. A P-value of less than 0.05 was considered significant. SVF, stroma-vascular fraction. AT, adipose tissue. (TIF) [file pone.0161739.s001.tif]
