# Supplementary material for: FTO Obesity Risk Variants Are Linked to Adipocyte IRX3 Expression and BMI of Children - Relevance of FTO Variants to Defend Body Weight in Lean Children?
Source: PLoS One. 2016 Aug 25;11(8):e0161739. doi: 10.1371/journal.pone.0161739 (PMC4999231; doi:10.1371/journal.pone.0161739)
Supplement: S1 Table — (DOC) [file pone.0161739.s002.doc]

**S1 TABLE.** Primer and probe sequences for quantitative *real-time* RT-PCR

| **Gene** | **Forward Primer** | **Reverse Primer** | **Probe** |
| --- | --- | --- | --- |
| *IRX3* | CTCTCCCTGCTGGGCTCT | CAAGGCACTACAGCGATCTG |  |
| *IRX5* | CGTTCTCGCCCTACGCT | GTAGGGAGAGCCCACGTA |  |
| *UCP1* | ACGACACGGTCCAGGAGTTC | ACCAGCTAAAATCTTGCTTCCTAAAC | TCACCGCAGGGAAAGAAACAGCACC |
| *TBP* | TTGTAAACTTGACCTAAGACCATTGC | TTCGTGGCTCTCTTATCCTCATG | AACGCCGAATATAATCCCAAGC GGTTTG |
| *ACTB* | TGAGCGCGGCTACAGCTT | CCTTAATGTCACGCACGATTT | ACCACCACGGCCGAGCGG |
| *HPRT* | GGCAGTATAATCCAAAGATGGTCAA | GTCTGGCTTATATCCAACACTTCGT | CAAGCTTGCTGGTGAAAAGGACCCC |

Forward and reverse primers are given in 5´-3´direction. Probes were labelled with the reporter 5’-FAM or 5’-HEX for *TBP* and the quencher 3’-TAMRA. *IRX3* and *IRX5* expression were determined using SYBR green.
