# Supplementary material for: FTO Obesity Risk Variants Are Linked to Adipocyte IRX3 Expression and BMI of Children - Relevance of FTO Variants to Defend Body Weight in Lean Children?
Source: PLoS One. 2016 Aug 25;11(8):e0161739. doi: 10.1371/journal.pone.0161739 (PMC4999231; doi:10.1371/journal.pone.0161739)
Supplement: S2 Table — (DOC) [file pone.0161739.s003.doc]

**S2 TABLE.** Correlations of adipocyte *IRX3* with obesity-related patient parameters unadjusted and adjusted for puberty stage

|  | unadjusted | | adjusted for PH | | |
| --- | --- | --- | --- | --- | --- |
| **Parameters** | r | *p* | r | *p* |  |
| BMI SDS | -0.265 | **0.016** | -0.219 | 0.059 |  |
| Aidpocyte diameter [µm] | -0.434 | **0.001** | -0.409 | **0.003** |  |
| Number of macrophages^a^ | -0.301 | **0.013** | -0.293 | **0.017** |  |
| CD68 expression^a^ | -0.428 | **<0.001** | -0.447 | **<0.001** |  |
| Serum adiponectin [mg/l]^a^ | 0.266 | **0.037** | 0.206 | 0.109 |  |
| Serum leptin [ng/ml]^a^ | -0.404 | **0.001** | -0.376 | **0.003** |  |
| HOMA-IR^a^ | -0.335 | **0.008** | -0.303 | **0.018** |  |

PH, pubic hair; BMI, body mass index; SDS, standard deviation score; HOMA-IR, homeostasis model assessment of insulin resistance. Significant *p-*values (*p*<0.05) are indicated in **bold**. ^a^Statistical analyses were performed for log-transformed parameters.
